# Comparative Transcriptome Analysis in Oilseed Rape (*Brassica napus*) Reveals Distinct Gene Expression Details between Nitrate and Ammonium Nutrition

**DOI:** 10.3390/genes10050391

**Published:** 2019-05-22

**Authors:** Weijie Tang, Xin He, Lunwen Qian, Feng Wang, Zhenhua Zhang, Chao Sun, Liangbin Lin, Chunyun Guan

**Affiliations:** 1 National Center of Oilseed Crops Improvement, Hunan Branch, Hunan Agricultural University, Changsha 410128, China; weijietangmr@163.com (W.T.); hexinzhsh@126.com (X.H.); qianlunwen@163.com (L.Q.); wangfenghifi@126.com (F.W.); zhzh1468@163.com (Z.Z.); 2 College of Agronomy and Biotechnology, Yunan Agricultural University, Kunming 650201, China; sunchaoynau@163.com; 3 Southern Regional Collaborative Innovation Center for Grain and Oil Crops in China, Hunan Agricultural University, Changsha 410128, China

**Keywords:** oilseed rape, nitrate, ammonium, physiology, transcriptome, gene expression

## Abstract

Nitrate (NO_3_^−^) and ammonium (NH_4_^+^) are the main inorganic nitrogen (N) sources absorbed by oilseed rape, a plant that exhibits genotypic differences in N efficiency. In our previous study, the biomass, N accumulation, and root architecture of two oilseed rape cultivars, Xiangyou 15 (high N efficiency, denoted “15”) and 814 (low N efficiency, denoted “814”), were inhibited under NH_4_^+^ nutrition, though both cultivars grew normally under NO_3_^−^ nutrition. To gain insight into the underlying molecular mechanisms, transcriptomic changes were investigated in the roots of 15 and 814 plants subjected to nitrogen-free (control, CK), NO_3_^−^ (NT), and NH_4_^+^ (AT) treatments at the seedling stage. A total of 14,355 differentially expressed genes (DEGs) were identified. Among the enriched Gene Ontology (GO) terms and Kyoto Encyclopedia of Genes and Genomes (KEGG) pathway categories of these DEGs, carbohydrate metabolism, lipid metabolism, protein metabolism, and cell wall biogenesis were inhibited by AT treatment. Interestingly, DEGs such as N transporters, genes involved in N assimilation and *CESA* genes related to cellulose synthase were also mostly downregulated in the AT treatment group. This downregulation of genes related to crucial metabolic pathways resulted in inhibition of oilseed rape growth after AT treatment.

## 1. Introduction

Nitrogen (N) is an indispensable macronutrient for plant growth and development that not only serves as a constituent of many crucial macromolecules, including proteins, enzymes, and nucleic acids, but also acts as a signal to regulate many biological processes from metabolism to resource allocation, growth and development. Nitrate (NO_3_^−^) and ammonium (NH_4_^+^) are the main N forms absorbed and utilized by plants, accounting for 70% of anion and cation absorption [1]. The mechanisms used by plants to absorb and utilize NO_3_^−^ and NH_4_^+^ differ, and NO_3_^−^ and NH_4_^+^ can affect plant growth and physiological processes, such as dry matter accumulation, root morphology, photosynthesis, and N assimilation. Accordingly, researchers have carried out studies on the effects of different N forms on the growth and physiology of plants, but the conclusions of these studies vary due to differences among plant species and genotypes. For example, some plants such as rice, tea, and *Vaccinium* spp. prefer NH_4_^+^ and grow better under NH_4_^+^ nutrition [2,3,4], whereas others such as wheat, watermelon, and *Arabidopsis thaliana* prefer NO_3_^−^ [5,6,7].

Oilseed rape is a crucial source of plant oil worldwide, with a large demand for N fertilizer, and judicious application of N fertilizer can significantly improve the yield of oilseed rape [8]. Furthermore, a certain amount of N can increase oilseed rape biomass and yield, though excessive N decreases the oil content of oilseed rape [9]. Moreover, different oilseed rape genotypes exhibit distinct differences in their absorption and utilization of N fertilizers [10]. In most dryland soils, NO_3_^−^ is the primary form of inorganic N; however, in natural environments, the N form may be highly variable, with NH_4_^+^ often predominant in acidic and/or water-logged soils [11,12]. Dryland and acidic soils are both suitable for planting oilseed rape. To date, NO_3_^−^ accumulation and utilization have been a major emphasis of studies on N use in oilseed rape. In contrast, less is known about the growth performance and gene expression of these plants under NH_4_^+^ nutrition and whether oilseed rape cultivars with different N efficiencies have the same or different reactions to NO_3_^−^ and NH_4_^+^ nutrition. Hence, we evaluated the effects of NO_3_^−^ and NH_4_^+^ on the growth and physiology of two oilseed rape cultivars with different N efficiencies. The results demonstrated that NH_4_^+^ greatly inhibited the growth and root architecture of both cultivars but that they grew and developed normally under NO_3_^−^ nutrition [13]. 

Although NO_3_^−^ and NH_4_^+^ clearly have distinct effects on the growth of the two cultivars, studies of oilseed rape under NO_3_^−^ and NH_4_^+^ nutrition at the molecular level are rare. Oilseed rape is closely related to *Arabidopsis*. Differences in gene expression in *Arabidopsis* under NO_3_^−^ and NH_4_^+^ nutrition have been explored using microarray analysis, and the results suggest that the underlying bases of nitrate- and ammonium-specific patterns of gene expression differ [14]. In general, comparative high-throughput RNA sequencing (RNA-Seq) analyses of different cultivars and treatments can provide a deep understanding of the actual metabolic pathways involved, and a rice transcriptomic encyclopedia has recently been used to explore a large number of ammonium-responsive genes [2]. Moreover, Yang et al. [15] explored the transcriptomic profiles of tea (*Camellia sinensis*) treated with different N forms using RNA-Seq. The development of these metabolic databases has greatly facilitated studies focusing on NO_3_^−^ and NH_4_^+^ nutrition. Thus, RNA-Seq appears to be a promising technique for determining the genomic behavior of oilseed rape under NO_3_^−^ and NH_4_^+^ nutrition.

Here, the effects of NO_3_^−^ and NH_4_^+^ application on the physiological responses of two oilseed rape cultivars, namely, Xiangyou 15 (with high N efficiency) and 814 (with low N efficiency), are explored. RNA-Seq of roots was applied to reveal molecular mechanistic differences between NO_3_^−^ and NH_4_^+^ nutrition. Some candidate genes associated with N uptake, transport and assimilation, enriched Gene Ontology (GO) terms and enriched metabolic pathways were identified to provide a valuable resource for further understanding the major transcriptomic regulatory pathways responding to NO_3_^−^ and NH_4_^+^ in the roots of two rape genotypes.

## 2. Materials and Methods

### 2.1. Plant Materials and Treatments

The two oilseed rape cultivars used in this study were previously characterized as having high (Xiangyou 15, denoted “15”) or low (814) N efficiency [16]. Seeds of 15 and 814 were provided by the National Center of Oilseed Crops Improvement, Hunan Branch, Hunan, China. Mature seeds were sown in trays with growth medium containing a mixture of peat and vermiculite (1:1, *v*/*v*). After producing ~3–4 leaves (35 d after sowing), we transplanted uniform seedlings into different pots for physiological and transcriptomic experiments. To prevent NH_4_^+^ from being converted into NO_3_^−^ by microorganisms, this study used pot culture (diameter: 30 cm; height: 30 cm) with quartz sand containing no nutrients. The nutrient solution used was based on the improved Hoagland nutrient solution [17].

The physiological experiment was conducted in 2016. All pots with different N treatments contained the same levels of N (15 mmol/L) and dicyandiamide (DCD) (10 μmol/L) to prevent NH_4_^+^ transformation. There were six treatments, i.e., 2 cultivars × 3 N treatments. Ten seedlings were used for each treatment, with only one seedling per pot, for a total of 60 seedlings. Table 1 shows the macronutrient composition of the hydroponic solution of the three N treatments. The micronutrient composition was as follows: B, 1.0 mg/L; Mn, 0.5 mg/L; Zn, 0.05 mg/L; Cu, 0.02 mg/L; and Mo, 0.01 mg/L. The NT + AT (75% NO_3_^−^ + 25% NH_4_^+^) treatment (Table 1) received a conventional oilseed rape fertilizer and was set as a control in the physiological experiment. The hydroponic solution was refreshed every five days. To prevent reaction between Ca_2_^+^ and CO_3_^2−^/SO_4_^2−^, CaCl_2_ was applied separately. The total N input of each treatment was 2.8 g/pot, and all N was added to pots before 60 d after transplanting. 

The transcriptome experiment was conducted in 2017. Most of the methods were similar to those used in the physiological experiment. The NT (100% NO_3_^−^) and AT (100% NH_4_^+^) treatments were the same as those in the physiological experiment. We used a nitrogen-free treatment (control, CK) instead of the NT + AT treatment to further distinguish the transcriptomic characteristics of oilseed rape roots in the NT and AT treatments. The 11.25 mmol/L KNO_3_, 1.875 mmol/L (NH_4_)_2_SO_4_ and 1.875 mmol/L K_2_CO_3_ (Table 1, NT + AT) treatments were replaced by 7.5 mmol/L K_2_CO_3_ (CK), and the other nutrients were the same. Twenty seedlings were included in each treatment, with only one seedling per pot, for a total of 120 seedlings. At 48 h after exposure to different N treatments, root tissues from five seedlings in each treatment group (three biological replicates) were collected, mixed, washed with double-distilled water, immediately frozen in liquid nitrogen, and stored at −80 °C for use in RNA extraction.

### 2.2. Measurement of Growth-Related Parameters

For biomass and N concentration determination, five plants at the bud period (70 d after transplanting) from each treatment were individually harvested and separated into shoots and roots. All the plant samples were washed with double-distilled water, heated at 105 °C for 30 min and dried at 80 °C until their weight remained constant; the dry weight (DW) was recorded. The N concentrations of the shoots and roots were determined using a SmartChem Discrete Auto Analyzer 200 (AMS Systea, Roma, Italy) with H_2_SO_4_-H_2_O_2_ digestion.

Five roots from each treatment were first separately cleaned with double-distilled water to remove the growth medium. Each sample was placed in a glass rectangular sink (300 mm × 200 mm × 20 mm) in batches with a layer of water approximately 6–8 mm deep to allow the roots to extend fully and minimize overlap for capturing images; each sample was scanned at 600 dpi with an automatic scanning apparatus (Perfection V700, Epson, Nagano, Japan). The total root length (sum of path length of the main and lateral roots), root surface area (sum of areas of the main and lateral roots), average root diameter (average diameter of the main and lateral roots), and root volume (sum of volumes of the main and lateral roots) were acquired by analysis of root images using WinRHIZO Pro 2009 software (Regent Instruments, Québec, QC, Canada).

### 2.3. RNA Quantification and Qualification

Total RNA was extracted manually from five mixed root tissues using a modified CTAB method [18] (three biological replicates) for a total of 15 root tissues per treatment. RNA integrity was assessed at 2.2 ≥ OD260/280 ≥ 1.8 using an RNA Nano 6000 Assay Kit with a Bioanalyzer 2100 system (Agilent Technologies, Palo Alto, CA, USA). RNA purity was checked using a NanoDrop spectrophotometer (Thermo Fisher, Waltham, MA, USA). RNA concentrations were measured using a Qubit RNA Assay Kit and a Qubit 3.0 Fluorometer (Life Technologies, Carlsbad, CA, USA). Equal amounts of total RNA from three biological replicates per condition were pooled to construct the cDNA library; the remaining RNA was carefully preserved for later use (quantitative real-time polymerase chain reaction, qRT-PCR). Sequencing was performed using the Illumina HiSeq X Ten platform and 150-bp paired-end module. The sequence data were deposited in the NCBI database and are accessible via BioProject ID: PRJNA518759.

### 2.4. Bioinformatic Analysis

The original image data obtained by high-throughput sequencing were converted to sequence data by CASAVA base calling. Each sample generated more than 8 gigabytes of data. For further analysis, the reads were filtered from raw sequencing data by removing adaptors and trimming low-quality (<Q30) reads. The processed reads were then mapped to the reference *Brassica* *napus* genome (http://brassicadb.org/brad/datasets/pub/Genomes/Brassica_napus/) [19], and transcript levels of unigenes were identified by HISAT2 (v2.0.5) [20,21]. The mapped reads for each sample were assembled using StringTie (v1.3.3) [20,22] with a reference-based approach. This method employs spliced reads to determine exon connectivity. 

Cuffdiff (v1.3.0) [23] was utilized to calculate the fragments per kilobase of exon per million fragments mapped (FPKMs) for coding genes in each sample. Gene FPKMs were computed by summing the FPKMs of the transcripts in each gene group, which were calculated based on the length of the fragments and the read count mapped to each fragment. Cuffdiff (v2.2.1) [23] provides statistical methods for determining differential expression in digital transcript or gene expression data sets using a model based on a negative binomial distribution. Significantly differentially expressed genes (DEGs) were identified with the following criteria: corrected *p*-value < 0.05 (false discovery rate (*FDR*) < 0.05) and |log_2_(fold change)| ≥ 1.

Gene functional annotations were based on *B. napus* (http://www.genoscope.cns.fr/brassicanapus/) and *Arabidopsis* (https://www.arabidopsis.org/index.jsp) genome databases and matched to GO terms (http://www.geneontology.org/). GO enrichment analysis was carried out using PerlModule (GO:: TermFinder) [24]. GO terms with a corrected *p*-value < 0.05 were considered to be significantly enriched among the DEGs. R functions (phyper and qvalue) were applied to test for statistical enrichment of DEGs among Kyoto Encyclopedia of Genes and Genomes (KEGG) pathways. KEGG pathways with a corrected *p*-value < 0.05 were considered to be significantly enriched among the DEGs.

### 2.5. Quantitative RT-PCR Analysis

To validate the differential expression patterns of genes obtained by Illumina sequencing, qRT-PCR was conducted for 15 genes using total RNA from three biological replicates per treatment and gene-specific primer pairs designed based on the target gene sequences using Primer Premier 5.0 software (Table S1). First-strand cDNAs were synthesized from 1 µg of total RNA using Revert Aid Premium Reverse Transcriptase (Thermo Scientific™, EP0733). Three independent biological replicates and three technical replicates of each biological replicate were used for real-time PCR analysis. The total reaction volume for each qRT-PCR was 20 µL, which comprised 10 µL of SybrGreen qPCR Master Mix, 0.8 µL of forward and reverse primers, 2 µL of 1:10-diluted cDNA and 7.2 µL of double-distilled water. The PCR conditions were as follows: 95 °C for 3 min followed by 45 cycles of 95 °C and for 3 s 60 °C for 30 s. The reactions were performed using an ABI StepOne Plus Real-Time PCR system. The *B. napus ACTIN7* gene-specific primer (Table S1) was employed as a control to normalize expression, and relative expression was calculated using the 2^−∆Ct^ × 1000 method [25]. 

### 2.6. Statistical Analysis

All statistical analyses of data from the experiment and qRT-PCR were performed with Microsoft Excel (Microsoft Corporation, Redmond, WA, USA) and SPSS 22.0 (SPSS Inc., Chicago, IL, USA). Differences among treatments were analyzed by one-way analysis of variance (ANOVA) and differences between cultivars by the t-test. Differences were considered significant at *p* < 0.05.

## 3. Results

### 3.1. Effects of Different N Treatments on the Growth of Two Oilseed Rape Cultivars

**Figure 1:**
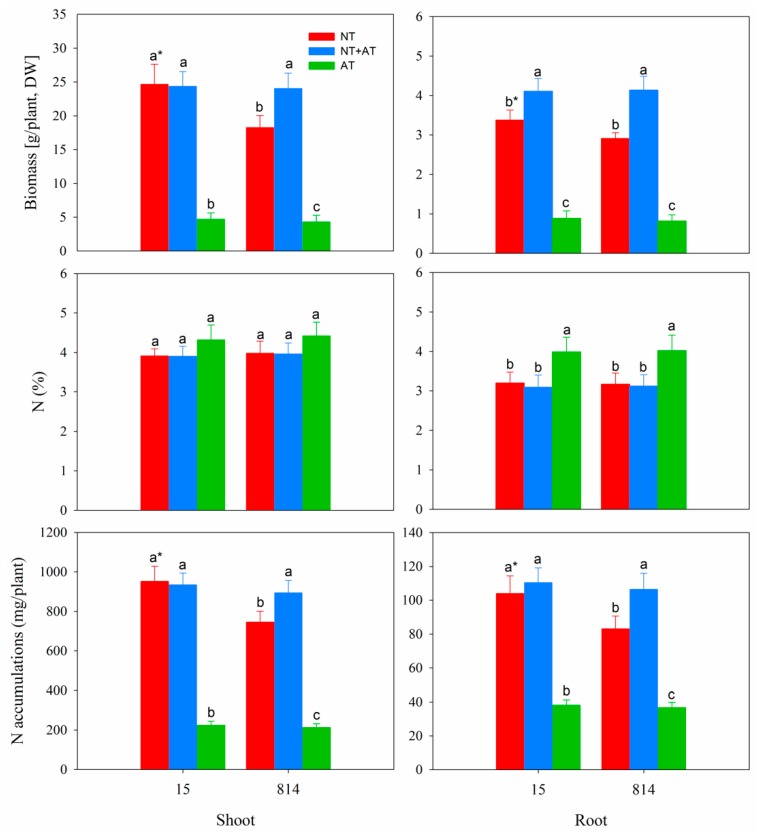
Growth performances of two cultivars, namely, 15 (high N efficiency) and 814 (low N efficiency), after 70 d in different N treatments. The three treatments contained the same amount of N (15 mmol/L). Different letters at the top of the histogram bars denote significant differences among treatments for the same cultivar (*p* < 0.05); asterisks (*) at the top of the histogram bars denote significant differences between cultivars in the same treatment (*p* < 0.05); vertical bars indicate the standard deviation (SD) (n = 5).

As shown in Figure 1, the DW of both 15 and 814 shoots and roots decreased dramatically when the plants were supplied with NH_4_^+^. Compared with NT + AT, the NT treatment significantly decreased the DW of both 15 and 814 roots and the DW of 814 shoots, with no significant change in the DW of 15 shoots. Compared with the NT treatment, AT decreased the shoot and root DW by 81% and 77% and by 74% and 72% in 15 and 814, respectively. Although N concentrations in shoots and roots in the AT treatment were higher than those in the NT and NT + AT treatments for both cultivars, N accumulation in shoots and roots in the AT treatment was much lower than that in the NT and NT + AT treatments. Therefore, a high concentration of NH_4_^+^ as the sole N source was unfavorable to oilseed rape growth, N absorption and accumulation compared to a high concentration of NO_3_^−^ as the sole N source. In addition, biomass and N accumulation of 15 shoots and roots in the NT treatment were significantly higher than those of 814 in this treatment.

**Figure 2:**
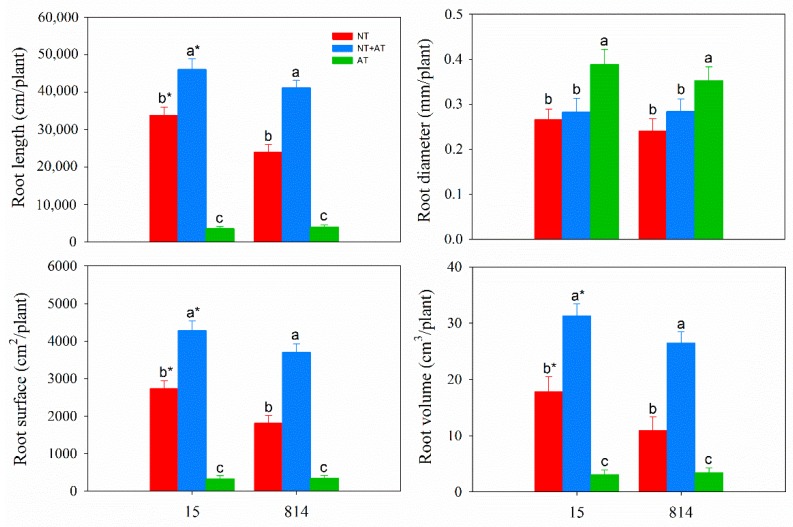
Root architecture of the two cultivars under different N treatments. The three treatments contained the same amount of N (15 mmol/L). Different letters at the top of the histogram bars denote significant differences among treatments for the same cultivar (*p* < 0.05); asterisks (*) at the top of the histogram bars denote significant differences between cultivars in the same treatment (*p* < 0.05); vertical bars indicate the SD (n = 5).

The root morphology of the two cultivars was greatly influenced by the NT and AT treatments (Figure 2). Of the three N treatments, NT + AT most increased the root length, root surface area, and root volume of the two cultivars, and these traits were greater in the NT treatment group than in the AT treatment group for the two cultivars. Compared with NT, AT reduced the root length, root surface area and root volume of 15 and 814 by 10.6%, 12.0%, and 17.3% and by 16.7%, 19.4%, and 31.2%, respectively. In contrast, the diameter in the AT treatment group was thicker than that in the NT and NT + AT treatment groups for both cultivars, though the root length, root surface area and root volume of 15 (with high N efficiency) were higher than those of 814 in the NT and NT + AT treatment groups. 

### 3.2. Summary of High-Throughput RNA Sequencing Data

In the transcriptomic experiment, cDNA from six samples was subjected to Illumina sequencing. The number of clean reads was 76,474,884, 74,236,004, 72,682,964, 69,526,330, 66,139,042, and 90,465,100 in 15-CK, 15-NT, 15-AT, 814-CK, 814-NT, and 814-AT, respectively, after trimming and filtering (Table S2); the total number of mapped reads was 89.72%, 83.44%, 89.10%, 89.20%, 83.44%, and 88.82%, respectively. The frequency of a >30 Phred quality score (Q30) was >92% and the guanine-cytosine (GC) content >46% for the six samples (Table S2). Approximately 83.44%–89.72% of the clean reads were successfully mapped to the *B. napus* genome (http://brassicadb.org/brad/datasets/pub/Genomes/Brassica_napus/) using TopHat2 software, and 65.05%–70.34% of the clean reads matched unique genomic locations (Table S2). In total, 57,749, 60,261, 56,295, 56,989, 58,473, and 57,842 genes were expressed in 15-CK, 15-NT, 15-AT, 814-CK, 814-NT, and 814-AT, respectively. These results suggested that the RNA-Seq data used in the present study were highly reliable.

### 3.3. Differentially Expressed Gene Analysis

**Figure 3:**
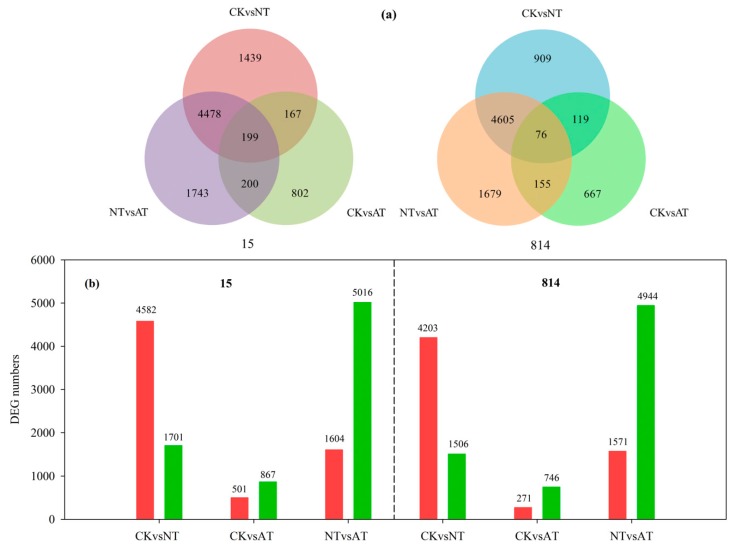
(**a**) A Venn diagram illustrating overlaps among differentially expressed genes (DEGs) in different comparison groups, i.e., CKvsNT, CKvsAT, and NTvsAT, in 15 and 814; (**b**) a bar chart showing up- and downregulated DEGs in the six comparison groups. The red column shows upregulated DEGs, and the green column shows downregulated DEGs. CK: control.

Using the six samples, we constructed six comparison groups, CKvsNT (15), CKvsAT (15), NTvsAT (15), CKvsNT (814), CKvsAT (814), and NTvsAT (814), and 14,355 unigenes (Table S3) were identified as significant DEGs in the six comparison groups, which contained 6283, 1368, 6620, 5709, 1017, and 6515 DEGs (Figure 3a), respectively. Two comparisons between the CK and NT treatments (15 and 814) revealed 4582 and 4203 upregulated and 1701 and 1506 downregulated DEGs in 15 and 814, respectively; 501 and 271 upregulated and 867 and 746 downregulated DEGs were found between the CK and AT treatments (15 and 814, respectively) (Figure 3b). Furthermore, compared with NT, the AT treatment resulted in many more downregulated DEGs, with 5016 and 4944 in 15 and 814, respectively (Figure 3b). Correlation of gene expression levels among samples is an important index for testing the reliability of experiments and the rationality of sample selection: the closer the correlation coefficient is to 1, the higher is the similarity between samples. Table S3 shows Pearson correlations for gene expression levels between different comparison groups. The Pearson correlation of gene expression levels between 15-CK and 15-NT was only 0.261, whereas that between 15-CK and 15-AT was as high as 0.876 (Table S4). As expected, the Pearson correlation between 814-CK and 814-NT was only 0.275, but that between 814-CK and 814-AT was as high as 0.946 (Table S4). This finding indicated fundamental differences in gene expression levels between the NT and AT treatments and that gene expression levels in oilseed rape roots in the AT treatment group were similar to those in the CK treatment group.

### 3.4. Gene Ontology Enrichment Analysis of Differentially Expressed Genes

**Figure 4:**
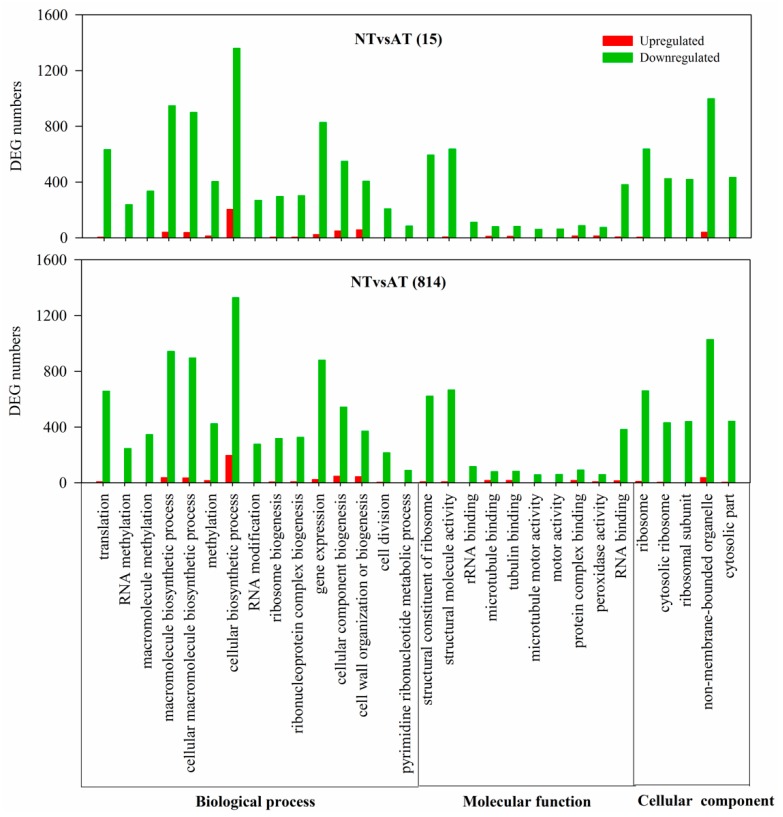
Enriched Gene Ontology (GO) terms for NTvsAT (15 and 814). The results are summarized under three top-level ontologies: biological process, molecular function, and cellular component. The *x*-axis represents the functional category of the DEGs. The *y*-axis indicates the number of annotated genes expressed in a given subcategory. The downregulated DEGs are represented by green, and the upregulated DEGs are represented by red.

GO enrichment analysis was performed to broadly classify all of the DEGs into corresponding biological process, molecular function, and cellular component categories. GO terms in each category were sorted from lowest to highest by corrected *p*-values. To understand the distinct response of the two genotypes to the NT and AT treatments, we analyzed the enriched GO terms of the comparison groups CKvsNT (15 and 814) (Figure S1) and CKvsAT (15 and 814) (Figure S2). There were more upregulated than downregulated DEGs in enriched GO terms for CKvsNT (15 and 814); more downregulated than upregulated DEGs were detected among enriched GO terms for CKvsAT (15 and 814). Moreover, the numbers of DEGs for CKvsNT (15 and 814) were much higher than those for CKvsAT (15 and 814). Furthermore, the number of downregulated DEGs among enriched GO terms was also greater than the number of upregulated DEGs for NTvsAT (15 and 814) (Figure 4). In addition, highly enriched GO terms for NTvsAT DEGs according to the biological process category are involved in translation, RNA methylation, and macromolecule methylation; those according to the molecular function category included structural constituent of ribosome, structural molecule activity, and rRNA binding predominated, those and according to the cellular component category included ribosome, cytosolic ribosome and ribosomal subunit. These results indicate distinctive differences in the transcript levels of certain genes between the NT and AT treatments.

### 3.5. Kyoto Encyclopedia of Genes and Genomes Metabolic Pathway Enrichment Analysis

**Figure 5:**
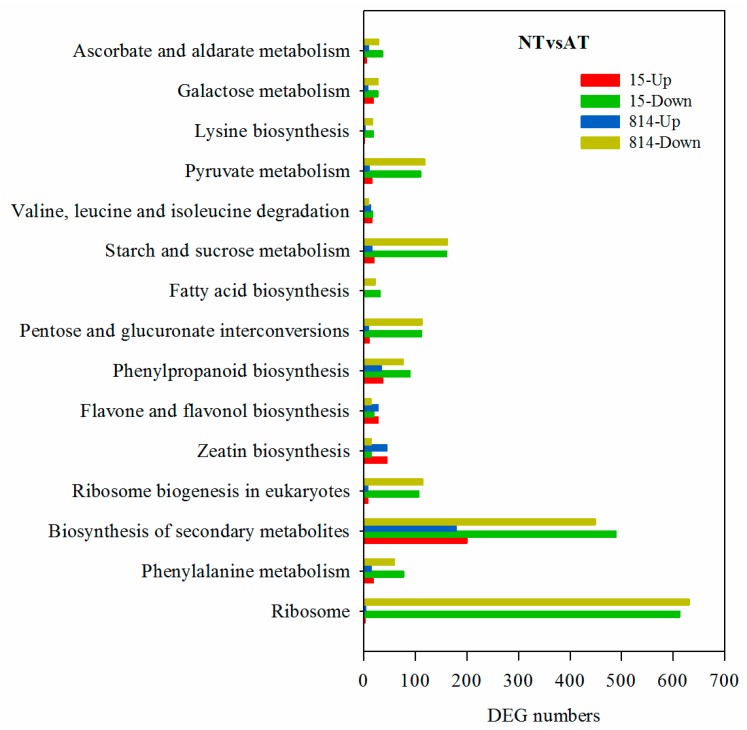
Top fifteen enriched Kyoto Encyclopedia of Genes and Genomes (KEGG) pathways for NTvsAT (15 and 814). The *x*-axis represents the number of DEGs involved in each pathway; the *y*-axis depicts pathways arranged from bottom to top based on the degree of enrichment.

Pathway enrichment analysis of DEGs using the KEGG database (http://www.genome.jp/kegg/) identified 122, 123, 118, 110, 124, and 124 KEGG pathways for the six comparison groups CKvsNT (15), CKvsNT (814), CKvsAT (15), CKvsAT (814), NTvsAT (15), and NTvsAT (814), respectively. The top 15 enriched pathways for the two genotypes contained more upregulated DEGs than downregulated DEGs for CKvsNT (Figure S3) but more downregulated DEGs than upregulated DEGs for CKvsAT (Figure S4). DEGs involved in ribosome, biosynthesis of secondary metabolites and phenylalanine metabolism were more enriched in CKvsNT, whereas DEGs involved in biosynthesis of secondary metabolites, phenylalanine metabolism and phenylpropanoid biosynthesis were more enriched in CKvsAT. In NTvsAT (Figure 5), there were more downregulated than upregulated DEGs, and DEGs involved in ribosome, phenylalanine metabolism and biosynthesis of secondary metabolites were highly enriched.

### 3.6. Genes Involved in Starch and Sucrose Metabolism, Fatty Acid Biosynthesis, Ribosome and Cell Wall Biogenesis

Carbohydrates, lipids and proteins are the main organic materials that compose plant cells and provide energy. In the pathway enrichment analysis in this study, we found three metabolic pathways, namely, starch and sucrose metabolism (SSM) (ko00500), fatty acid biosynthesis (FAB) (ko00061) and ribosome (RIB) (ko03010), which are the primary pathways of carbohydrate metabolism, lipid metabolism and translation, respectively, to be enriched in CKvsNT and NTvsAT. DEGs involved in SSM, FAB, and RIB showed the same trend. The numbers of DEGs involved in SSM, FAB, and RIB in CKvsNT (15 and 814) and NTvsAT (15 and 814) were greater than those in CKvsAT (15 and 814) (Table 2). Moreover, there were more upregulated DEGs in SSM, FAB, and RIB and fewer downregulated DEGs in the NT treatment group than in the CK treatment group, despite few differences in response to the AT treatment. There were fewer upregulated DEGs in SSM, FAB, and RIB and more downregulated DEGs in the AT treatment group than in the NT treatment group. Information on the DEGs involved in SSM, FAB, and RIB in the six comparison groups is provided in Table S5. To further determine whether the molecular levels of carbohydrates, lipids and proteins were significantly altered in the different N treatments, we examined the numbers of up- and downregulated DEGs for each pathway in carbohydrate metabolism, lipid metabolism and translation in the six comparison groups (Table S6). The results indicate that carbohydrate metabolism, lipid metabolism and protein metabolism were inhibited in the AT treatment group, which led to decreased growth.

The cell wall is the major component of plant roots. In the GO term analysis performed in this study, we found that the numbers of DEGs involved in primary cell wall biogenesis (CWB) (GO: 0009833) differed between the comparison groups (Table 2). There were 8 and 6 upregulated DEGs in CKvsNT (15 and 814), 9 and 5 downregulated DEGs in NTvsAT (15 and 814) and, interestingly, no DEGs in CKvsAT (15 and 814). Furthermore, DEGs associated with CWB are members of the cellulose synthase (*CESA*) gene superfamily, including *CESA1*, *CESA2*, *CESA3*, and *CESA6* (Table S5). Therefore, NT treatment enhanced the transcriptional levels of genes related to CWB, whereas AT treatment had no effect on primary CWB.

### 3.7. Genes Related to N Uptake, Transport, and Assimilation

**Figure 6:**
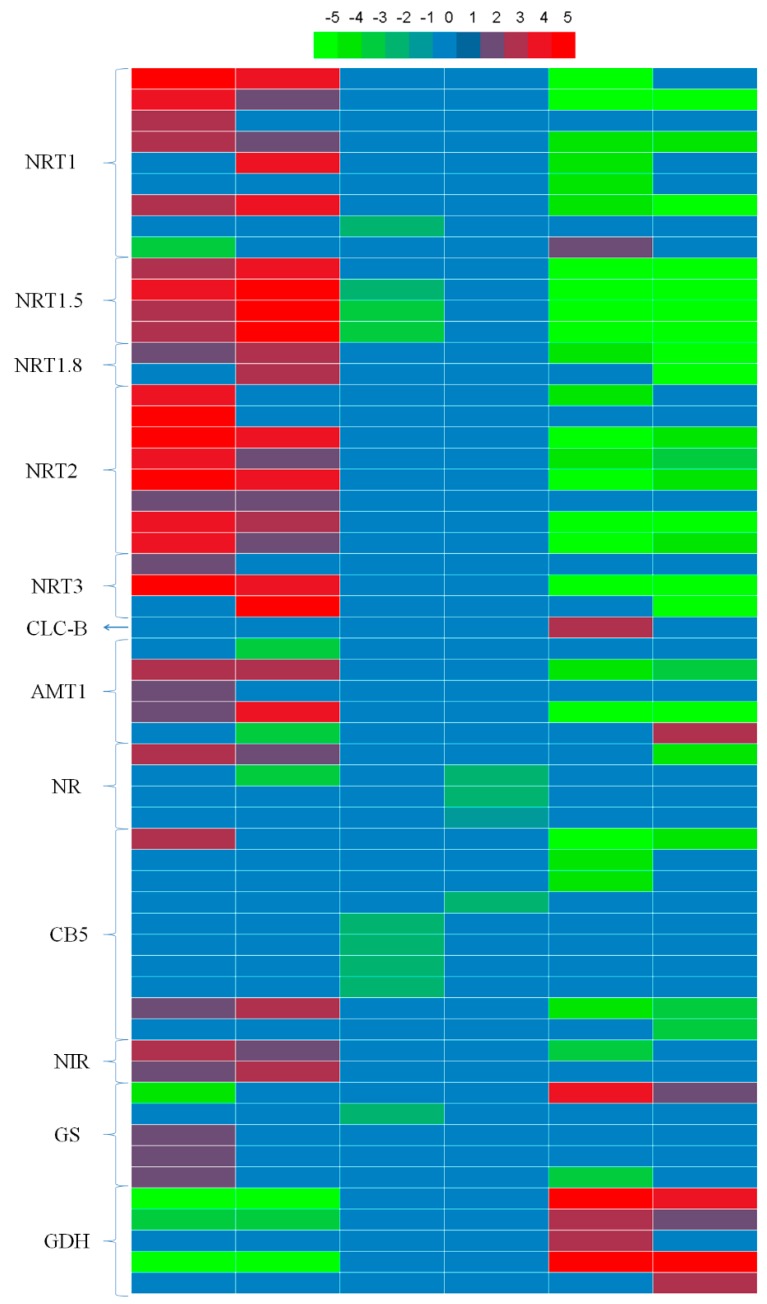
Heatmap showing the relative expression patterns of genes related to N metabolism between different comparison groups in 15 and 814. From left to right: 15-log_2_(NT/CK), 814-log_2_(NT/CK), 15-log_2_(AT/CK), 814-log_2_(AT/CK), 15-log_2_(NT/AT), and 814-log_2_(NT/AT). Colors indicate the relative differential gene expression: green, downregulated; red, upregulated; and blue, not differentially expressed.

N metabolism in plants mainly involves three processes: N uptake, transport and assimilation. Many genes involved in NO_3_^−^/NH_4_^+^ uptake, transport and assimilation were differentially expressed in the six samples (Tables S2 and S6). In the current study, 27 DEGs encoding NO_3_^−^ transporters (*NRT1*, *NRT1.5*, *NRT 1.8*, *NRT2*, *NRT3*, and *CLC-B* (chloride channel protein B)) were detected (Figure 6, Table S7). The abundances of the transcripts of these genes, except for 1 DEG (BnaC09g20910D), were increased in CKvsNT; transcript levels of 4 genes decreased in CKvsAT (15). In NTvsAT, the transcript levels of most DEGs were reduced in both 15 and 814, but 2 DEGs (BnaC09g20910D and BnaC02g36720D) were upregulated only in 15. A total of 5 genes encoding NH_4_^+^ transporters were identified, of which BnaCnng01740D was responsive only in 15 among the six samples (Table S7). Transcript levels of 3 DEGs were increased in CKvsNT (15). Two DEGs were upregulated in CKvsNT (814), though the other 2 DEGs were downregulated (Figure 6). One DEG (BnaA01g23190D) was upregulated in NTvsAT (814). Two DEGs were downregulated in NTvsAT (15 and 814). 

Some of the DEGs encode key enzymes in NO_3_^−^/NH_4_^+^ assimilation, including 4 DEGs encoding nitrate reductase (*NR*), 10 DEGs encoding cytochrome B5 (*CB5*) of *NR*, 2 DEGs encoding nitrite reductase (*NIR*), 5 DEGs encoding glutamine synthetase (*GS*), and 5 DEGs encoding glutamate dehydrogenase (*GDH*) (Figure 6, Table S7). In CKvsNT, there were more upregulated DEGs than downregulated DEGs encoding *NR*, *CB5*, *NIR*, and *GS* in the two cultivars. In CKvsAT, there were more downregulated DEGs than upregulated DEGs encoding *NR*, *CB5* and *GS* in the two cultivars. In NTvsAT, 1 DEG encoding *NR*, 5 DEGs encoding *CB5*, and 1 DEG encoding *NIR* were downregulated; 1 upregulated DEG and 1 downregulated DEG encode *GS1.3* and *GS2*, respectively (Table S7). Interestingly, DEGs encoding *GDH* were always downregulated in CKvsNT and upregulated in NTvsAT.

### 3.8. Validation of Genes Using Quantitative Real-Time PCR

**Figure 7:**
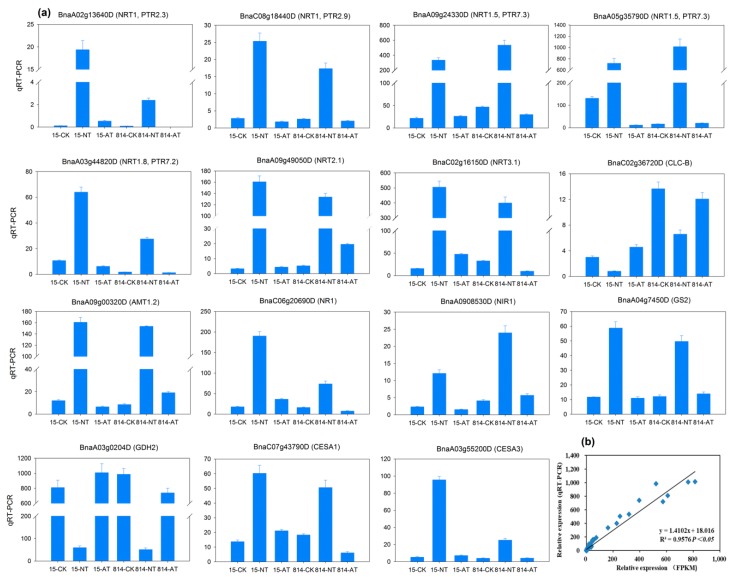
Quantitative real-time PCR (qRT-PCR) validation of 15 differentially expressed genes (DEGs). (**a**) Transcript levels of 15 DEGs. Vertical bars indicate SDs (n = 3). The columns represent relative expression obtained by qRT-PCR. (**b**) Comparison of relative expression obtained from RNA-Seq data and qRT-PCR. RNA-Seq relative expression (*x*-axis) is plotted against qRT-PCR relative expression (*y*-axis). The correlation coefficient (R^2^) is indicated in the figure. FPKM: fragments per kilobase of exon per million fragments mapped.

To confirm the accuracy and reproducibility of the Illumina RNA-Seq results, 15 representative genes were chosen based on the above analysis, and their expression levels in 15 and 814 in the different N treatment groups were validated by quantitative real-time PCR (qRT-PCR) (Figure 7a). The 15 representative genes encoded low- and high-affinity NO_3_^−^/NH_4_^+^ transporters, *NR*, *NIR*, *GS*, *GDH*, and catalytic subunits of cellulose synthase, which are key proteins or enzymes in N metabolism and root development. Among these DEGs, the qRT-PCR profiles for the 15 representative genes mostly agreed with those obtained from RNA-Seq, with minimal differences in expression (Figure 7a). Pearson correlation (coefficient 0.9576) revealed strong agreement between the qRT-PCR data for these genes and the RNA-Seq results (Figure 7b). Therefore, the RNA-Seq data presented herein are reliable.

## 4. Discussion

NO_3_^−^ and NH_4_^+^ are the main N sources for oilseed rape, but studies investigating the effects of NO_3_^−^ and NH_4_^+^ on the physiology and transcriptome levels of oilseed rape are scarce. Our primary goal in this work was to differentiate the physiological and molecular effects of the two most common sources of N utilized by oilseed rape: NO_3_^−^ and NH_4_^+^. There were clear, distinct differences in physiology and transcriptome levels between NO_3_^−^ and NH_4_^+^ nutrition.

### 4.1. Oilseed Rape Seedling Growth Was Inhibited by AT treatment

In the physiological experiment, compared with a high concentration of NO_3_^−^ as the sole N source, a high concentration of NH_4_^+^ significantly reduced oilseed rape biomass, root growth, and N accumulation. These results indicate that a high concentration of NH_4_^+^ as the sole N source is a source of stress for oilseed rape but that NO_3_^−^ or NO_3_^−^ plus a small amount of NH_4_^+^ is a suitable N nutrition environment for growth. Some studies have found that NO_3_^−^ plus a small amount of NH_4_^+^ is beneficial for the growth of crops that prefer NO_3_^−^ [6] and that NH_4_^+^ plus a small amount of NO_3_^−^ is beneficial for the growth of crops that prefer NH_4_^+^ [26]. Additionally, some studies have confirmed that a single application of NH_4_^+^ or overapplication of NH_4_^+^ is conducive to the growth of most dryland crops [5,6]. As NO_3_^−^ taken up by plant cells can be stored in large quantities in vacuoles, this form of N is non-toxic for plants. NO_3_^−^ concentrations in plant cell vacuoles and the cytoplasm range 30–50 mol/m^3^ and 3–5 mol/m^3^ [27,28], respectively, whereas NH_4_^+^ is rarely found in vacuoles. Indeed, NH_4_^+^ taken up by plant cells must be combined with organic acids as soon as possible, leading to the synthesis of amino acids or amides and the acidification of cells. Recent studies have suggested that NH_4_^+^ can restrain uptake of K^+^, Ca^2+^, Mg^2+^, and other cations by crops [6]. A high concentration of NH_4_^+^ can also suppress the activities of key enzymes in crop N metabolism, such as *NR*, *GS*, and *GOGAT* (Glutamate synthase) [29]. Therefore, a high concentration of NH_4_^+^ is likely to result in NH_4_^+^ toxicity in dryland crops. In summary, oilseed rape appears to be a nitrate-preferring crop.

The N efficiency of a crop has two meanings. On the one hand, it refers to the amount of N absorbed by crops at the same N supply level; on the other hand, it refers to high use efficiency of the N absorbed and the amount of dry matter produced per unit of N absorbed [30]. In this experiment, compared with the N-inefficient cultivar 814, the N-efficient cultivar 15 displayed better root architecture and higher N use efficiency under the NT treatment. The root system is the main organ for absorbing water and nutrients and is important for organic matter assimilation, transformation, and synthesis [31]. The 15 cultivar showed a better root length, root surface, and root volume than did 814, which led to more N uptake and accumulation in 15 than in 814 under the NT treatment. Our previous work [16] showed that compared with the N-efficient cultivar 15, the N-inefficient cultivar 814 stores more NO_3_^−^ absorbed by the root system in root vacuoles, resulting in a decrease in NO_3_^−^ transported from the root system to the aboveground part. However, to meet the growth and developmental needs of the aboveground part, the root system of the 814 cultivar needs to absorb more NO_3_^−^. In other words, the 814 cultivar must absorb more NO_3_^−^ to produce the same biomass as 15, which leads to lower N use efficiency in the former.

GO (Figure S6) and KEGG (Figure S7) analyses of DEGs between 15 and 814 at 48 h after NT treatment (15vs814(NT)) revealed some differences in gene expression between the two cultivars, especially DEGs related to the cell wall (Table S8) and N metabolism (Table S9). Overall, 1877 DEGs (734 upregulated and 1143 downregulated) were identified (Figure S5). GO analysis showed high enrichment of DEGs involved in the organonitrogen compound metabolic process, oxidoreductase activity, catalytic activity and the cell wall, which was consistent with a recent report using 15 and 814 as test materials treated with low NO_3_^−^ (0.30 mm/L) for 0 h, 3 h, and 72 h [32]. The expression levels of both *BnaA02g13640D* (*NRT1*) and *BnaA01g26960D* (*NRT1*), which are responsible for efficient NO_3_^−^ uptake, were higher in the roots of 15 than in 814 in our study. In contrast, mRNA levels of *BnaA09g47380D* (*NRT1.1*) and *BnaC08g43380D* (*NRT2.1*) were much higher in the roots of 15 than in 814 in the recent report [32], particularly under low NO_3_^−^. Because the N solution concentration, N treatment time and seedling period were different between our study and the previous report, we conclude that the discrepancy may be attributed to gene expression being affected by the growth period and N treatment. In previous hydroponic and sand culture experiments, we found no significant differences in phenotypes (growth and physiology indexes) between 15 and 814 under NT treatment during the early seedling period (0–50 d after seedling emergence). Therefore, to analyze the transcriptomic results between 15 and 814 under NT treatment at different growth periods, it is helpful to comprehensively understand the molecular mechanism of N efficiency differences in oilseed rape.

Although a high concentration of NH_4_^+^ as the sole N source is detrimental to oilseed rape growth at the physiological level, the related transcriptomic changes remain largely unknown. In our study, we applied RNA-Seq to evaluate gene expression under different N treatments in the roots of seedlings of the two genotypes and examined many of the genes that were differentially expressed among the CK, NT and AT treatments. These genes are mainly involved in N metabolism, CWB, and carbohydrate, lipid and protein metabolism.

### 4.2. N Metabolism of Oilseed Rape Was Inhibited by AT treatment

Compared with CK, most genes involved in N metabolism were markedly elevated by the NT treatment, though these genes were not induced by the AT treatment. The latest transcriptomics results for N limitation (0.3 mmol/L NO_3_^−^) in oilseed rape show that genes related to N uptake and allocation, such as *BnaNRT1.1 s* (*B. napus* nitrate transporter *1.1 s*), *BnaNRT1.4 s*, *BnaNRT1.5 s*, *BnaNRT1.9 s*, *BnaNRT2.1 s*, *BnaNRT2.4 s*, and *BnaNRT3.1 s*, are induced in roots by N limitation, and N metabolism genes such as *BnaGS1s* and *BnaGS2s* are also elevated in roots under N limitation; in contrast, genes such as *BnaNRT1.8 s*, *BnaNIA1s*, and *BnaNIA2s* are downregulated in roots [33]. These results are not consistent with our findings. The majority of genes related to N metabolism were induced in the roots of oilseed rape exposed to N limitation, whereas these genes were mostly strongly repressed in roots by the N-free (CK) treatment. This might be caused by the different adaptive mechanisms of oilseed rape in response to N limitation or N-free conditions. Overall, increased N uptake and transport and enhanced assimilation of inorganic N into amino acids might be helpful for the adaptability of oilseed rape to N limitation [33].

**Figure 8:**
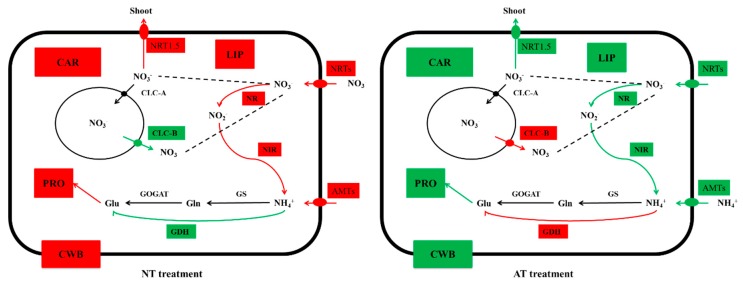
A proposed model for N metabolism and other primary metabolism in oilseed rape roots in NT and AT treatment groups. The colored rectangles indicate the following: red, N transporters, enzymes or physiological processes that increased; and green, N transporters, enzymes or physiological processes that decreased. Red or green solid arrows represent the strengthening or weakening of physiological processes, and black solid arrows represent physiological processes that did not significantly change. Compared with AT treatment, NT treatment led to upregulation of the *NRT/AMT* genes in roots and facilitated NO_3_^−^ uptake. Simultaneously, *NR* and *NIR* activities increased, and *BnNRT1.5* gene expression was upregulated, which facilitated more NO_3_^−^ transport into shoots from roots for assimilation under NT treatment. This enhanced transport was beneficial for oilseed rape growth because NO_3_^−^ assimilation efficiency is known to be higher in shoots than in roots due to the utilization of solar energy by oilseed rape shoots. Furthermore, carbohydrate metabolism (CAR), lipid metabolism (LIP), protein metabolism (PRO), and CWB were promoted by NT treatment, which facilitated oilseed rape root growth and development. Thus, NT treatment was more beneficial to oilseed rape growth than was AT treatment.

NT treatment enhanced expression of *BnNRTs* but AT treatment decreased that of *BnAMTs* in oilseed rape. Four types of transporters, namely, low- and high-affinity NO_3_^−^ transporters (*NRTs*)/NH_4_^+^ transporters (*AMTs*), take up and reallocate NO_3_^−^ and NH_4_^+^ in angiosperms, respectively [34]. NO_3_^−^ induction of *GmNRT2* mRNA expression was accompanied by a fourfold increase in net NO_3_^−^ uptake by soybean roots under 100 μM external NO_3_^−^ [35]. In addition, correlation coefficients between ^13^NO_3_^−^ influx from 100 μM and 5 mM [NO_3_^−^] suggest that in *A. thaliana*, high- and low-affinity transport systems are mediated primarily by *AtNRT2.1* and *AtNRT1.1*, respectively [36]. In our physiology experiment, N accumulation in the NT treatment group was significantly higher than that in the AT treatment group. Therefore, upregulated expression of most *BnNRTs* under NT treatment greatly promoted NO_3_^−^ uptake by oilseed rape. Recent research has demonstrated that downregulation of *AtAMT1* genes, which mediate the majority of NH_4_^+^ uptake in *Arabidopsis* roots, is associated with decreased NH_4_^+^ accumulation in the *AtNRT1.1* mutants *chl1-1* and *chl1-5* under conditions of high NH_4_^+^ [29]. The adjustment of oilseed rape to excessive NH_4_^+^ conditions via downregulation of the transcript levels of *BnAMTs* might be an adaptive mechanism (Figure 8) for reducing NH_4_^+^ uptake. Other research also supports this mechanism. For example, ^13^NH_4_^+^ influx and transcript levels of *OsAMT1.3* in roots decreased within 48 h when plants acclimated to 10 µm of external NH_4_^+^ for 3 weeks were transferred to 10 mm NH_4_^+^ [37].

NT treatment increased expression of *BnNRT1.5* while decreasing that of *BnCLC-B*. Long-distance transport and distribution of NO_3_^−^ between roots and shoots are regulated by two genes encoding components involved in long-transport mechanisms. *NRT1.5* is responsible for NO_3_^−^ xylem loading, and *NRT1.8* is responsible for NO_3_^−^ xylem unloading [38,39]. *BnNRT1.5* expression in the roots of oilseed rape increased significantly with NO_3_^−^ treatment, driving greater NO_3_^−^ long-distance transport from roots to shoots [16], which was beneficial for oilseed rape growth because NO_3_^−^ assimilation efficiency is known to be higher in shoots than in roots due to solar energy utilization by shoots [40]. The NT treatment resulted in overexpression of all three *NRT1.5* genes in both genotypes (Tables S3 and S7), indicating that more NO_3_^−^ was transported to the shoot from the root for assimilation under NT. Thus, the NO_3_^−^ assimilation efficiency of oilseed rape was promoted by the NT treatment. *AtCLC-B*, one of seven *AtCLC* transport proteins in *A. thaliana* that are responsible for vacuolar NO_3_^−^ short-distance transport and are the main channels for NO_3_^−^ movement between vacuoles and the cytosol, conducts strong outwardly rectifying anionic currents that are greatest in the presence of NO_3_^−^ [16,41,42]. Highly expressed BnaC02g36720D (*BnCLC-B*) most likely led to increased transport of NO_3_^−^ from vacuoles into the cytosol to satisfy the demand for NO_3_^−^ in the AT treatment group (Figure 6 and Figure 8). This result further verified that oilseed rape is a nitrate-preferring crop. 

NT treatment led to upregulation of more genes encoding *NR*, *CB5*, and *NIR* than did AT treatment. NO_3_^−^ reduction involves concerted reactions in which NO_3_^−^ is reduced to nitrite (NO_2_^−^) by *NR* and NO_2_^−^ is reduced to NH_4_^+^ by *NIR* [43]. Each *NR* subunit contains 3 prosthetic groups, namely, FAD, heme (*CB5*) and a molybdenum cofactor (MoCo) [44]. As previously reported for many higher plant species, transcriptional and nitrate-inducible regulation is one of the major mechanisms of *NR* activity in rapeseed [45]. Previous studies have also demonstrated that nitrate-supplied *Arabidopsis* roots exhibit large increases in the abundance of *NR* (At1g77760), *NR* (At2g15620) and *NADH-GOGAT* (At5g53460) transcripts and a >10-fold increase in *NR* enzyme activity [14]. Hence, our results are consistent with the above results.

Furthermore, *BnGDH2* was specifically induced by the AT treatment (Figure 6 and Figure 8). *GDH* may play a major role in maintaining a subtoxic NH_4_^+^ level in the cytoplasm [46] and might operate via amination. *GDH* responds positively to abiotic stress and is important for the detoxification of NH_4_^+^ under stress [47,48,49]. In addition, transcriptomic analysis of *Arabidopsis* roots supplied with NO_3_^−^ or NH_4_^+^ demonstrated that *AtGDH2* is specifically induced by NH_4_^+^ [14], which is consistent with our results. Hence, we can conclude that oilseed rape roots endured the excessive but subtoxic NH_4_^+^ level in the cytoplasm under AT treatment by upregulating the transcript levels of *BnGDH2* (Figure 8), which further suggests that a high concentration of NH_4_^+^ tends to result in NH_4_^+^ toxicity in oilseed rape roots at the transcriptome level.

### 4.3. Expression Levels of Genes Involved in Cell Wall Biogenesis Decreased with AT treatment

In this work, the root length, root surface area and root volume in the NT treatment group were significantly greater than those in the AT treatment group, which demonstrated that AT inhibited root development. All plant cells are surrounded by an extensible primary cell wall, which contains cellulose, pectin and noncellulosic polysaccharides [50]. Additions and rearrangements of cell wall components are required throughout growth and development, such as for cell elongation and cell proliferation in developing roots [51]. The *AtCESA* gene superfamily, which encodes the catalytic subunits of cellulose synthase, has been identified in hundreds of seed plant species and well characterized in *Arabidopsis* [52]. Plants have evolved complex regulatory mechanisms, including expression of *CESA* genes and modification of *CESA* proteins, to control cellulose biosynthesis and assembly in cell walls [53]. In this work, genes related to primary CWB (GO: 0009833) (Figure 8) were differentially expressed under the different N treatments, including *BnCESA1*, *BnCESA2*, *BnCESA3*, and *BnCESA6*, which encode cellulose synthase family proteins (Table S4). In *A. thaliana, AtCESA1* (*A. thaliana* cellulose synthase *1*), *AtCESA3*, and *AtCESA6* are preferentially expressed in expanding tissues [54], but *AtCESA2* is poorly understood. The NT treatment greatly increased the transcript levels of *BnCESA1*, *BnCESA2*, *BnCESA3*, and *BnCESA6*, whereas the AT treatment had no distinct effects (Table S5, Figure 8). This difference might constitute one line of evidence explaining why AT treatment inhibited root development in oilseed rape in this study.

In summary, there were significant differences between NO_3_^−^ and NH_4_^+^ nutrition with regard to biomass, N accumulation and root architecture in oilseed rape cultivars. The potential molecular mechanisms may be explained by most of the downregulated genes being related to N metabolism, cell wall biosynthesis, and carbohydrate, lipid, and protein metabolism in oilseed rape exposed to a high concentration of NH_4_^+^. To the best of our knowledge, this is the first comprehensive transcriptomic analysis of oilseed rape grown under NO_3_^−^ and NH_4_^+^ conditions, and the findings provide valuable resources for better understanding the responses of oilseed rape to NO_3_^−^ and NH_4_^+^ and subsequent improvement of N utilization efficiency.

## 5. Conclusions

In the present study, the biomass, N accumulation and root architecture of two oilseed rape cultivars with different N efficiencies were significantly and analogously influenced by NO_3_^−^ and NH_4_^+^ nutrition. To reveal the molecular mechanisms responsible for the differences in the two cultivars in response to CK, NO_3_^−^, and NH_4_^+^ conditions, RNA-Seq of root samples collected at 48 h after exposure to different N treatments was performed to analyze gene expression patterns related to N metabolism and other crucial metabolic pathways in plants. NH_4_^+^ directly decreased the biomass, N accumulation, and root architecture of the two oilseed rape cultivars and affected the overall metabolism of shoots and roots; this result was related to downregulated expression of NO_3_^−^/NH_4_^+^ transporters or channels and crucial metabolic pathways (including carbohydrate, lipid, and protein metabolism) in the two oilseed rapes. The root expression levels of *CESA* genes encoding cellulose synthase decreased under NH_4_^+^ nutrition, inhibiting root growth. These results provide insight into the regulatory mechanisms of some candidate genes, which will reveal the molecular mechanisms of decreased oilseed rape growth in response to a high concentration of NH_4_^+^ as the sole N source.

## Figures and Tables

**Table 1:** Macronutrient composition of the hydroponic solution.

Treatment	Nutrient Concentration (mmol/L)						
	KNO_3_	(NH_4_)_2_SO_4_	K_2_CO_3_	CaCl_2_	MgSO_4_	KH_2_PO_4_	Fe-EDTA
NT	15	0	0	2	2	1	0.2
NT + AT	11.25	1.875	1.875	2	2	1	0.2
AT	0	7.5	7.5	2	2	1	0.2

NT, 100% NO_3_^−^ treatment; NT + AT, 75% NO_3_^−^ + 25% NH_4_^+^ treatment; AT, 100% NH_4_^+^ treatment.

**Table 2:** Numbers of up- and downregulated genes in starch and sucrose metabolism (SSM), fatty acid biosynthesis (FAB), ribosome (RIB), and cell wall biogenesis (CWB) in the six comparison groups.

Comparison Groups	SSM		FAB		RIB		CWB	
	Up	Down	Up	Down	Up	Down	Up	Down
CKvsNT(15)	158	20	28	1	481	1	8	0
CKvsNT(814)	142	13	21	1	566	1	6	0
CKvsAT(15)	18	20	3	1	6	29	0	0
CKvsAT(814)	4	8	0	2	0	9	0	0
NTvsAT(15)	19	161	0	32	2	613	0	9
NTvsAT(814)	16	162	0	22	4	631	0	5

## Supplementary Materials

The following are available online at https://www.mdpi.com/2073-4425/10/5/391/s1, Figure S1: Enriched Gene Ontology terms in comparison group CKvsNT (15 and 814). Figure S2: Enriched Gene Ontology terms in comparison group CKvsAT (15 and 814). Figure S3: Top fifteen enriched Kyoto Encyclopedia of Genes and Genomes (KEGG) pathways in CKvsNT (15 and 814). Figure S4: Top fifteen enriched Kyoto Encyclopedia of Genes and Genomes (KEGG) pathways in CKvsAT (15 and 814). Figure S5: Numbers of up- and downregulated DEG and fold change were analyzed in 15vs814 (NT). Figure S6: Enriched Gene Ontology (GO) terms in 15vs814 (NT). Figure S7: Top fifteen enriched Kyoto Encyclopedia of Genes and Genomes (KEGG) pathways in 15vs814 (NT). Table S1: List of primer sequences used for qRT-PCR analysis. Table S2: Summary of transcriptomic data. Table S3: Information for the 14,355 significantly differentially expressed genes (DEGs) detected in the six cDNA libraries. Table S4: Pearson correlation of gene expression levels between different comparison groups. Table S5: Information on the differentially expressed genes (DEGs) involved in starch and sucrose metabolism, fatty acid biosynthesis, ribosome and plant-type primary cell wall biogenesis in the six comparison groups. Table S6: Numbers of up- and downregulated genes in each pathway involved in carbohydrate metabolism, lipid metabolism and translation in the six comparison groups. Table S7: Genes encoding NO_3_^−^/NH_4_^+^ protein transporters and assimilation enzymes differing in expression between genotypes in response to different nitrogen treatments. Table S8: Numbers of up- and downregulated DEGs related to cell wall in 15vs814(NT). Table S9: Important DEGs related to N uptake, transport and assimilation in 15vs814(NT).
