# Supplementary material for: Comparative Transcriptome Analysis in Oilseed Rape (Brassica napus) Reveals Distinct Gene Expression Details between Nitrate and Ammonium Nutrition
Source: Genes (Basel). 2019 May 22;10(5):391. doi: 10.3390/genes10050391 (PMC6562433; doi:10.3390/genes10050391)
Supplement: Supplementary file 1 [file genes-10-00391-s001.zip › Supplementary files/Supplemental Figure and Table.docx]

**Supplemental Figure and Table**

**Supplemental Figure S1.** Enriched Gene Ontology terms in comparison group CKvsNT (15 and 814). The results are summarized for the three top-level ontologies: biological process, molecular function, and cellular component. The *x*-axis represents the functional category of the differentially expressed genes (DEGs). The *y*-axis indicates the number of annotated genes expressed in a given subcategory. The downregulated DEGs are represented by green, and the upregulated DEGs are represented by red.

**Supplemental Figure S2.** Enriched Gene Ontology terms in comparison group CKvsAT (15 and 814). The results are summarized under the three top-level ontologies: biological process, molecular function, and cellular component. The *y*-axis indicates the number of annotated genes expressed in a given subcategory. The downregulated DEGs are represented by green, and the upregulated DEGs are represented by red.

**Supplemental Figure S3.** Top fifteen enriched Kyoto Encyclopedia of Genes and Genomes (KEGG) pathways in CKvsNT (15 and 814). The *x*-axis represents the number of differentially expressed genes (DEGs) involved in the fifteen pathways; the *y*-axis depicts pathways arranged from bottom to top based on the degree of enrichment.

**Supplemental Figure S4.** Top fifteen enriched Kyoto Encyclopedia of Genes and Genomes (KEGG) pathways in CKvsAT (15 and 814). the *y*-axis depicts pathways arranged from bottom to top based on the degree of enrichment.

**Supplemental Figure S5.** Numbers of up- and downregulated DEGs and fold change were analyzed in 15vs814 (NT). The downregulated DEGs are represented by green, and the upregulated DEGs are represented by red.

**Supplemental Figure S6.** Enriched Gene Ontology (GO) terms in 15vs814 (NT). The downregulated DEGs are represented by green, and the upregulated DEGs are represented by red.

**Supplemental Figure S7.** Top fifteen enriched Kyoto Encyclopedia of Genes and Genomes (KEGG) pathways in 15vs814 (NT).

**Supplemental Table S1.** List of primer sequences used for qRT-PCR analysis.

**Supplemental Table S2.** Summary of transcriptomic data.

**Supplemental Table S3.** Information for the 14355 significantly differentially expressed genes (DEGs) detected in the six cDNA libraries. The gene name, fragments per kilobase of exon per million fragments mapped (FPKM) values in the six libraries and annotated information of homologs in *Arabidopsis thaliana* are presented in this table.

**Supplemental Table S4.** Pearson correlation of gene expression levels between different comparison groups.

**Supplemental Table S5.** Information on the differentially expressed genes (DEGs) involved in starch and sucrose metabolism, fatty acid biosynthesis, ribosome and plant-type primary cell wall biogenesis in the six comparison groups. The gene name, fragments per kilobase of exon per million fragments mapped (FPKM) values, and log2 fold change (LFC) in the different comparison groups and annotated information of homologs in *Arabidopsis thaliana* are presented in this table.

**Supplemental Table S6.** Numbers of up- and downregulated genes in each pathway involved in carbohydrate metabolism, lipid metabolism and translation in the six comparison groups.

**Supplemental Table S7.** Genes encoding NO_3_^-^/NH_4_^+^ protein transporters and assimilation enzymes differing in expression between genotypes in response to different nitrogen treatments. Blanks in the table indicate no significant difference in gene expression.

**Supplemental Table S8.** Numbers of up- and downregulated DEGs related to cell wall in 15vs814 (NT).

**Supplemental Table S9.** Important DEGs related to N uptake, transport and assimilation in 15vs814 (NT).
